# Pituitary enlargement in patients with cerebrospinal fluid drainage due to ventricular shunt insertion: know the condition and do not mistake for adenoma

**DOI:** 10.1007/s11102-022-01296-y

**Published:** 2023-01-18

**Authors:** Agnieszka Grzywotz, Yan Li, Nicole Unger, Cordula Kiewert, Witold X. Chmielewski, Ulrich Sure, Anne Uerschels, Karsten Wrede, Ilonka Kreitschmann-Andermahr

**Affiliations:** 1 Department of Neurosurgery and Spine Surgery, University Hospital Essen, University of Duisburg-Essen, Hufelandstr. 55, 45147 Essen, Germany; 2 Institute of Radiology and Neuroradiology, University Hospital Essen, University of Duisburg-Essen, Hufelandstr. 55, 45147 Essen, Germany; 3 Department of Endocrinology, Diabetes and Metabolism, University Hospital Essen, University of Duisburg-Essen, Hufelandstr. 55, 45147 Essen, Germany; 4 Department of Pediatric Endocrinology, University Hospital Essen, University of Duisburg-Essen, Hufelandstr. 55, 45147 Essen, Germany

**Keywords:** Pituitary enlargement, Pituitary hyperplasia, Shunt insertion, CSF pressure, ICP

## Abstract

**Objective:**

Childhood hydrocephalus patients treated by ventriculo-peritoneal (v.-p.) shunting are sometimes referred years after this therapy for evaluation of suspicious pituitary enlargement. Since pituitary size has been shown to depend on cerebrospinal fluid (CSF) pressure, we assume this phenomenon to be caused by shunt overdrainage. Therefore, we studied pituitary size and morphology in shunted hydrocephalus patients with radiological signs of high CSF drainage.

**Patients and methods:**

Retrospective study of pituitary size and morphology in 15 shunted patients with non-tumoral hydrocephalus and 7 shunted hydrocephalus patients due to childhood brain tumor compared to a population mean. In five brain tumor patients also pre- and postsurgical comparisons were performed.

**Results:**

Pituitary mid-sagittal size and pituitary volume were significantly higher in both hydrocephalus groups, compared to the population mean (midsagittal size t = 5.91; p < 0.001; pituitary volume, t = 3.03; p = 0.006). In patients available for pre- and postoperative comparison, there was also a significant increase in pituitary size and volume postoperatively (mean preoperative midsagittal height 2.54 ± 1.0 mm vs. 6.6 ± 0.7 mm post-surgery; mean pre-operative pituitary volume 120.5 ± 69.2 mm^3^ vs. 368.9 ± 57.9 mm^3^ post-surgery).

**Conclusion:**

Our results confirmed a significant increase in pituitary size and volume, mimicking pituitary pathology, after v.-p. shunt insertion. This phenomenon can be explained by the Monro–Kellie doctrine, stating that intracranial depletion of CSF—as caused by v.p. shunting—leads to compensatory intracranial hyperemia, especially in the venous system, with the consequence of engorged venous sinuses, most likely responsible for enlargement of the pituitary gland.

## Introduction

The U.S. American neurosurgeon Harvey Cushing is well known for his meticulous research and treatment in the field of pituitary pathologies. It is less well known, that he also made important contributions to the treatment of congenital and childhood-acquired hydrocephalus, a condition known since antiquity and, until some decades ago, associated with a dismal prognosis. He was the first to note, that cerebrospinal fluid (CSF) was produced by the choroid plexus [1] and proposed a variety of methods for temporary and permanent CSF drainage in his young hydrocephalic patients [2] However, up to the 1950s, all developed shunt systems were hampered by a high failure rate, mostly due to insufficient implant materials [3]. With the combined invention of artificial valves and silicone for shunt catheters around 1960, a worldwide therapeutic breakthrough in hydrocephalus treatment was achieved [3]. Many patients with insufficient CSF drainage were all of a sudden enabled to survive this formally lethal condition and lead independent lives, albeit at the cost of a myriad of complications such as shunt infection or disconnection [4]. Moreover, especially before the advent of programmable valves, many of the implanted shunt systems led to CSF overdrainage, a condition that can remain clinically asymptomatic or become apparent by orthostatic headache, pain or stiffness of the neck, nausea, diplopia, or, at worst, the development of subdural hematomas [5].

Working in a large neurosurgical department, we made the experience on several occasions that patients with hydrocephalus treated by ventriculo-peritoneal (v.-p.) shunting in childhood or young adulthood were referred years later to our department for neurosurgical evaluation of suspected pituitary pathology such as pituitary adenoma. However, we found this tentative diagnosis, usually raised first by the radiologists, who evaluated MRI images of these shunted patients, not to be accompanied by any other clinical, biochemical or radiological signs (apart from pituitary enlargement) supporting the diagnosis of pituitary neoplasia. We, therefore, assumed the diagnosis of adenoma or other pituitary pathology to be a diagnostic pitfall, supported by the observation that the pituitary gland has been shown to change in size in different states of CSF pressure [6]. In order to look into this matter more systematically, we conducted a retrospective, explorative study of pituitary size and morphology in patients with hydrocephalus and radiological markers of high CSF drainage treated after birth or in childhood by v.-p. shunting.

## Method

We performed an exploratory search of the magnetic resonance imaging (MRI) report database of the Institute of Radiology and Neuroradiology of the University Hospital Essen for keywords relating to shunt insertion and radiological markers of high CSF drainage such as v.-p. shunt, slit ventricles, and/or (pachy)meningeal Gadolinium enhancement [7] for a 10 years’ time period from 2012 to 2022. Of the retrieved radiology reports, the majority related to multiple investigations of a limited number of patients. The respective imaging investigations of the individual patients were screened and those with limited interpretation capacity of the pituitary gland were discarded. Measurement of pituitary gland size was performed on mid-sagittal and coronal images, using always the greatest extension in the respective plane. Pituitary volume was estimated using the formula: V = antero-posterior dimension × craniocaudal dimension × transverse dimension × 0.52 as described in [8]. Clinically relevant data of the patients were extracted from chart records.

In five brain tumor patients, in whom repeated imaging investigations before and after shunt insertion were available, we measured the size of the pituitary before, shortly after and at least 1 year after shunt insertion. In those patients who received their v.-p. shunt shortly after birth, mostly only individual follow-up MRIs were achieved in the database which were used for measurement.

## Statistics

All statistical analyses were conducted with SPSS 27. Descriptive statistics are presented as mean, standard error of mean (SEM) and range. For comparative analyses, the normal distribution of data was controlled for with the Shapiro–Wilk test. Independent t-test were used to compare the patients’ pituitary measures to an averaged age-matched population mean, which we calculated from [8] by averaging the pituitary measurements available for different age ranges based on the number of patients in the respective age ranges. Non-normally distributed data were compared with Mann–Whitney U tests.

## Results

### Patient characteristics

A total of 20 patients could be identified by our radiology report search. Two further patients fulfilling those criteria were identified during a routine visit of the neurosurgical outpatient department (both referred for suspected pituitary adenoma), resulting in a total of 22 patients (15 female, 7 male; mean age 22.3 ± 2.3 years) available for further investigation. They were divided into two patient groups: The first comprised 8 female and 7 male non-tumoral hydrocephalus patients (called non-tumor hydrocephalus group; NTHG). The diagnoses leading to shunt insertion were postmenigitic (n = 1) or posthemorrhagic hydrocephalus after premature birth (n = 5), hydrocephalus due to meningomyelocele with or without Chiari malformation (n = 5), other connatal malformations with hydrocephalus (n = 3) and suspected aqueductal stenosis with ventricular enlargement in one patient. Their age at shunt insertion was about 0 years (= shunt insertion in the first weeks after birth) except for two patients, who received their shunts at age of 8 and 21 years. Only one of the NTHG patients was implanted with an adjustable shunt valve, the other patients had received either medium pressure (n = 10) or high pressure (n = 1) valves. In three patients the valve type was not clearly identifiable on lateral radiographs. Theses valves had been implanted, however, before the era of programmable valves.

The second patient group was made up of a total of 7 (6 female, 1 male) patients with occlusive hydrocephalus due to childhood brain tumor (4 pilocytic astrocytomas and 3 medulloblastomas), here called tumor hydrocephalus group (THG). Their mean age of shunt insertion was 9.3 years (range 2–15 years). In all of these patients, non-adjustable medium pressure valves had been implanted. In five of the childhood cancer survivors (CCS) in the THG, endocrinological work-ups had been performed as part of childhood oncology surveillance protocols, in the remaining two endocrinological assessment was missing. Only one CCS patient developed post treatment hypopituitarism during the follow-up period, necessitating thyreotrophic and somatotrophic hormone replacement. In one of the NTHG patients, endocrinological investigation was prompted by significant pituitary enlargement following shunt insertion with unremarkable results. Apart of the CCS patient with partial hypopituitarism, none of the other patients in the THG received any hormone replacement or had clinical evidence of hormonal dysfunction.

Four of the investigated patients had clinical signs of CFS overdrainage, which manifested in all cases as orthostatic headache.

## Pituitary morphology, size and volume

Across all investigated patients, pituitary surface was convex in 16 (10 in the NTHG and 6 in the THG) and planar in six (5 in the NTHG and 1 in the THG). In none of the patients, indirect signs of pituitary adenoma such as a deviation of the pituitary stalk or bony enlargement/arrosion of bony sella and adjacent bone structures were present. In the NTHG, midsagittal pituitary height was 8.9 ± 1.7 mm (range 6.3–12.6 mm) and pituitary volume 460.3 ± 133.4 mm^3^ (range 260.8–644.0 mm^3^). In the THG patients, postsurgical pituitary midsagittal height and volume amounted to 8.2 ± 0.9 mm, range 6.1–13.2 mm and 411.5 ± 55.2 mm^3^, range 231.8–679.5 mm^3^, respectively. Pituitary size and volume did not statistically differ between the NTHG and the THG (all Z ≤ − 1.48, all p ≥ 0.138) (see Table 1).

**Table 1:** Pituitary morphology, size and volume in the NTHG versus THG

Patient group	Midsagittal pituitary height (mean ± SEM)	Pituitary volume (mean ± SEM)	Z-value	p-value
NTHG (n = 15)	8.9 ± 1.7 mm Range 6.3–12.6 mm	460.3 ± 133.4 mm^3^ Range 260.8–644.0 mm^3^		
THG (n = 7)	8.2 ± 0.9 mm Range 6.1–13.2 mm	411.5 ± 55.2 mm^3^ Range 231.8–679.5 mm^3^	− 1.48	0.138

Mann–Whitney U comparison of midsagittal pituitary height and pituitary volume of the NTHG vs. THG. Mean, SEM, range, Z-values and p-values are reported

Moreover, in five patients in the THG, pre- and postoperative MRIs could be analyzed. In this small subgroup, pituitary surface was concave before brain tumor treatment and shunt implantation, but planar or convex in the follow-up MRIs. Pituitary measurements preoperatively were 2.54 ± 1.0 mm, range 1.4–4.2 mm (pre-operative midsagittal height) and 120.5 ± 69.2 mm^3^, range 82.3–230.7 mm^3^ (pre-operative pituitary volume) as compared to 6.6 ± 0.7 mm, range 5.8–7.7 mm (post-operative midsagittal height) and 368.9 ± 57.9 mm^3^, range 329.5–465.7 mm^3^ (post-operative pituitary volume) 1 year after surgery. This difference was statistically significant (Z = − 2.02, p = 0.043) (see Table 2).

**Table 2:** Pituitary morphology, size and volume pre- and postoperatively in the THG

Patient group (n = 5)	Midsagittal pituitary height (mean ± SEM)	Pituitary volume (mean ± SEM)	Z-value	p-value
THG pre-operatively	2.54 ± 1.0 mm Range 1.4–4.2 mm	120.5± 69.2 mm^3^ Range 82.3–230.7 mm^3^		
THG postoperatively	6.6 ± 0.7 mm Range 5.8–7.7mm	368.9 ± 57.9 mm^3^ Range 329.5 –465.7 mm^3^	− 2.02	0.043

Mann–Whitney U test comparison of midsagittal pituitary height and pituitary volume of the THG (pre- vs. post-operatively). Mean, SEM, range, Z-values and p-values are reported

**Fig. 1:**
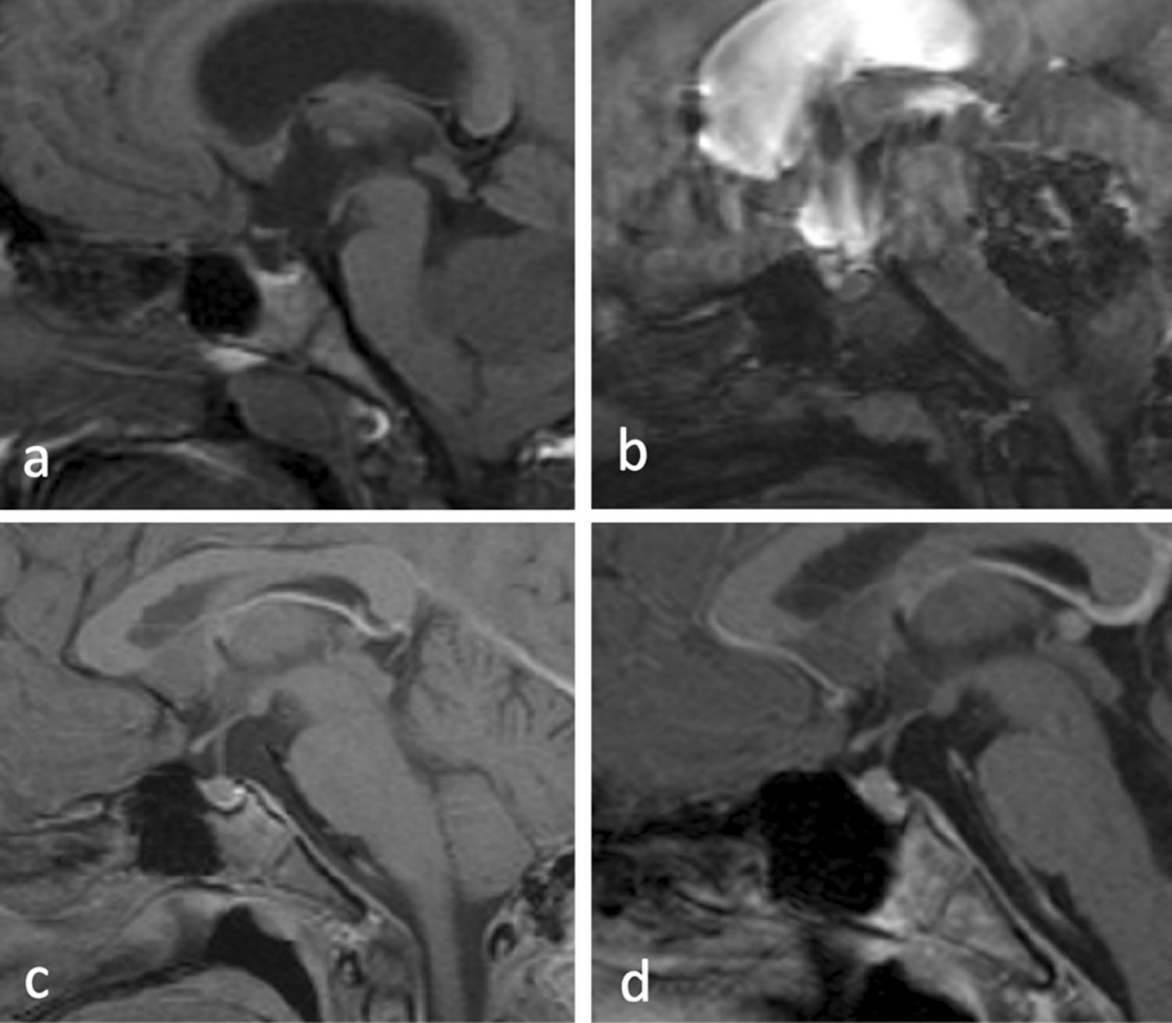
Time course of pituitary size in a child with a medulloblastoma: **a** preoperatively: note the partial empty sella due to increased intracranial pressure caused by obstructive hydrocephalus. **b** One day after tumor removal: note the mild increase in pituitary size despite the suboptimal scan quality due to movement artefacts. **c** 10 months after shunt insertion: note the development of convex pituitary surface. **d** 3.5 years after shunt insertion: note the further change in the height of the pituitary and slight change in sellar floor morphology

The two remaining CSS were not included in this comparison, as only postoperative MRIs after shunt insertion were available. Figure 1 shows the course of pituitary size in a THG patient before shunting, immediately postoperatively and after 1 and 4 years.

**Fig. 2:**
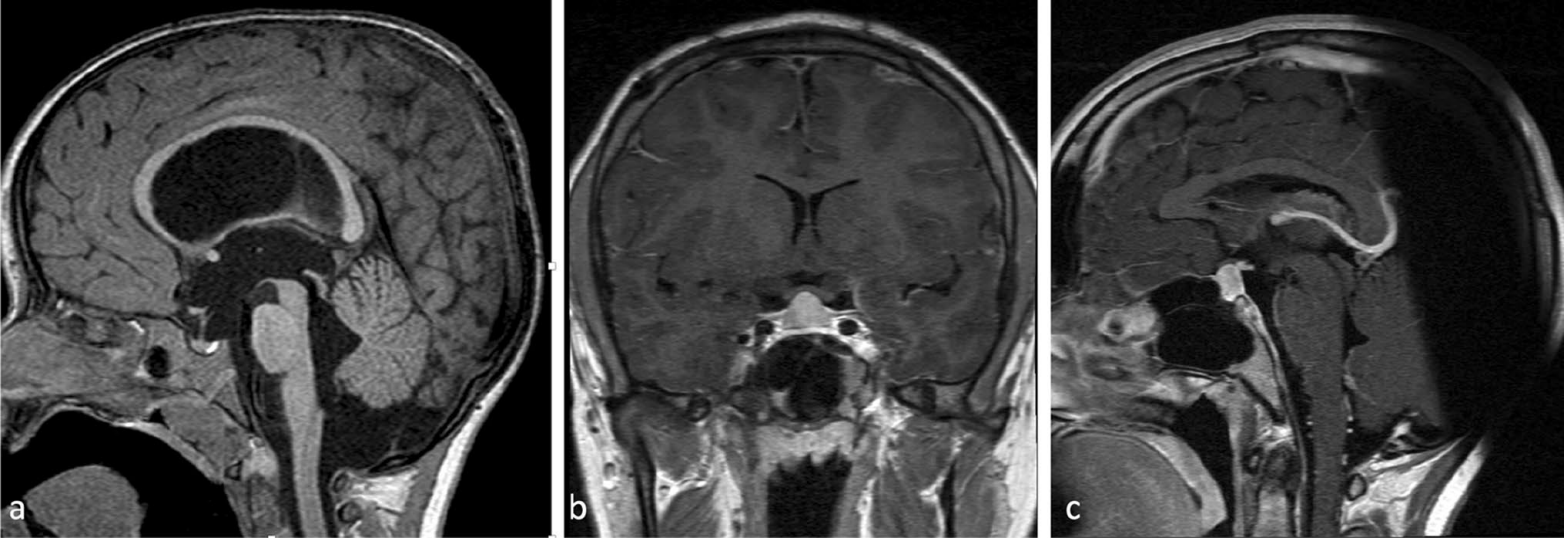
Change in pituitary morphology before and after shunt insertion in a patient with triventricular hydrocephalus of unknown etiology. **a**–**c** Note the change in ventricular and pituitary size before and more than 10 years after shunt insertion. On image **b** you can see the slight deformation of the optic chiasm caused by pituitary enlargement, while image **c** also shows a change in sellar floor morphology as compared to **a**

**Fig. 3:**
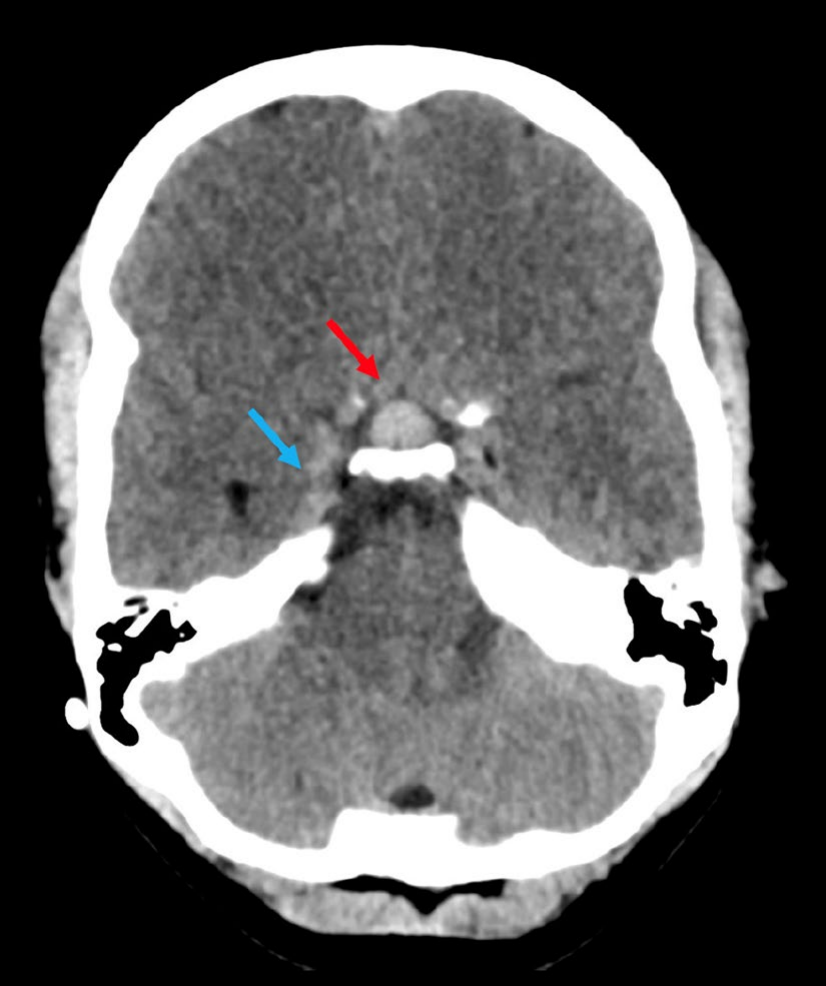
CT scan of the same patient as in Fig. 2. Note the pituitary hyperemia as illustrated by the slightly hyperdense aspect of the gland on CT scan (red arrow) and the engorgement of the cavernous sinus (blue arrow)

Since pituitary size and volume did not statistically differ between the NTHG and the THG, the comparison with the published age-matched healthy population mean was performed with the entire patient cohort (mean midsagittal height 8.5 ± 0.3 mm, range 6.1–13.2 mm, mean pituitary volume 444.8 ± 29.0 mm^3^, range 231.8–679.5 mm^3^). As expected, pituitary height (t = 5.91; p < 0.001) and volume (t = 3.03; p = 0.006) in the shunted hydrocephalus patients differed (highly) significantly to the averaged population mean [8]. Mean pituitary height and volume of those patients with clinical signs of CSF overdrainage did not differ significantly from those without (all Z ≤ − 1.56, all p ≥ 0.119). Figure 2 shows the change of pituitary morphology in a NTHG patient, while Fig. 3 illustrates the changed sellar floor morphology and pituitary hyperemia.

## Discussion

Our exploratory analysis confirmed our hypothesis of significant increase in pituitary size and volume, mimicking pituitary pathology, after v.-p. shunt insertion in children and adolescents with radiological signs of shunt overdrainage in comparison to the published population mean. In a small subgroup of brain tumor patients shunted for obstructive hydrocephalus, we could also demonstrate a change in pituitary morphology changing from a concave surface before treatment to planar or convex thereafter.

Up to now, pituitary hyperplasia occurring in the wake of v.-p. shunt insertion has been described infrequently in the literature. One Finnish group reported an increased pituitary size in children and adolescents with shunted hydrocephalus as compared to age and sex-matched normal controls which was associated with enhanced gonadotropin secretion [9]. However, based on this association and the observation of accelerated pubertal development in patients with shunted hydrocephalus [10], they postulated pituitary hyperplasia to be caused by “central stimulation, possibly hypothalamic in origin”, but did not provide any further explanations for this effect. On the contrary, other studies reported precocious puberty and amenorrhea to be the consequence of increased intracranial pressure in chronic untreated hydrocephalus and that CSF shunting led to a normalization of gonadotroph axis function (for an overview see [11]). A further argument speaking against increased gonadotropin secretion to be the cause of increased pituitary size in shunted patients can be found in the results published by van Beek et al. in 2000 [12], showing that no significant change occurred in any pituitary size or shape parameter following gondadotropin releasing hormone analogue therapy.

In another case study, highlighting the potential effects of chronic CSF overdrainage on skull base structures, the authors postulated that their patient’s pituitary was not truly enlarged, but that such an impression was given as the consequence of overdrainage-induced shrinkage of the sella turcica and subsequent upward extrusion of the pituitary gland [13]. Yet our results show, that a true and significant increase of pituitary size and volume occurs after v.-p. shunt insertion, while acknowledging from own unpublished observations that bony changes of the sella may additionally occur in patients with long-standing shunt overdrainage (for examples confer to Figs. 1 and 2).

In adults, pituitary hyperplasia has frequently been described in spontaneous intracranial hypotension (SIH), a condition characterized by a loss of CSF due to leakage through the dural membrane, i.e., at the level of the cervicothoracic spine, and classically accompanied by severe orthostatic headache [14, 15]. According to a recent study, this phenomenon of pituitary enlargement constitutes the most frequent early radiological sign of SIH, present in 97.6% of 42 investigated patients 1–6 days after symptom onset [16]. On the other end of the spectrum, a flattened pituitary (empty sella or partial empty sella) is recognized as one diagnostic marker in patients with benign intracranial hypertension, a condition of increased CSF production and consecutively elevated ICP, which can lead to chronic headaches and visual loss [17].

The altered size of the pituitary in relation to CSF pressure is best explained by the so-called Monro–Kellie doctrine, a hypothesis stating that the sum of the volumes of the brain, CSF and intracranial blood content is always constant due to their encasement by the rigid skull [18, 19]. An increase in one of the volumes should, thus be followed by a decrease in one or both of the remaining two. Since the brain volume remains nearly constant in states of decreased CSF volume, a compensatory intracranial hyperemia occurs, primarily in the venous system, as reflected by engorgement of venous sinuses and diffuse venous meningeal hyperemia [19]. In terms of the pituitary, this means that any drop in CSF—be it caused by shunting or spontaneous loss as in SIH—should incur an increase of blood volume in the ample, widely anastomosed arterial and sinusoidal venous blood supply of this gland [20] with the consequence of pituitary enlargement. On the other hand, an increase of ICP mediated by disturbance of CSF resorption or outflow, as in malresorptive or occlusive hydrocephalus, should lead to a decrease in the blood supply of the pituitary, making the soft, endocrine tissue more vulnerable for compression.

In sum, we showed a significant pituitary enlargement after v.-p. shunt insertion in children and adolescents in comparison to the age-related population mean. Together with the findings published in the literature we believe this enlargement to be primarily mediated by the drop in intracranial pressure after shunting, as exemplified by the radiological marker of slit ventricles. However, since this was a retrospective analysis with some patients lost to follow-up, endocrinological assessment to definitely rule out end organ failure or increased gonadotropin secretion as alternative explanations for the observed pituitary enlargement was not available in all patients. There was, however, no clinical evidence of severe untreated hypothyroidism in any of the investigated patients, that would have explained the pituitary hyperplasia that occurred in temporal relation with shunt insertion.

Next to the retrospective design and the predefined inclusion criterion of presence of slit ventricles as radiological markers of shunt overdrainage, an additional drawback of the present study is the relatively small sample size, which necessitated the calculation of normative estimates to explore pituitary parameter differences in comparison to the normal population and limits the generalizability of the present results. Future studies with larger patient samples and a prospective design, allowing for pre-post shunt comparisons and a stratification by clinical (i.e., age of shunt insertion, duration of treatment) and technical parameters (such as shunt system and valve pressure) are needed to confirm and further differentiate the present results. Moreover, it would be very interesting to investigate, whether neuroradiological aspects of shunt overdrainage such as pituitary morphology will regress after adjustment of shunt valve pressure. Already now, we can say that physicians involved in the diagnosis and therapy of patients with childhood hydrocephalus should know the potential consequences of this treatment on the pituitary gland and be aware of the entity of shunt-induced pituitary enlargement, not to be confused with pituitary adenoma.

## Data Availability

The anonymized dataset is available from the corresponding author at due request.
